# Supplementary figures and images for: Shedding light on dark figures: Steps towards a methodology for estimating actual numbers of COVID-19 infections in Germany based on Google Trends
Source: PLoS One. 2022 Oct 26;17(10):e0276485. doi: 10.1371/journal.pone.0276485 (PMC9605024; doi:10.1371/journal.pone.0276485)

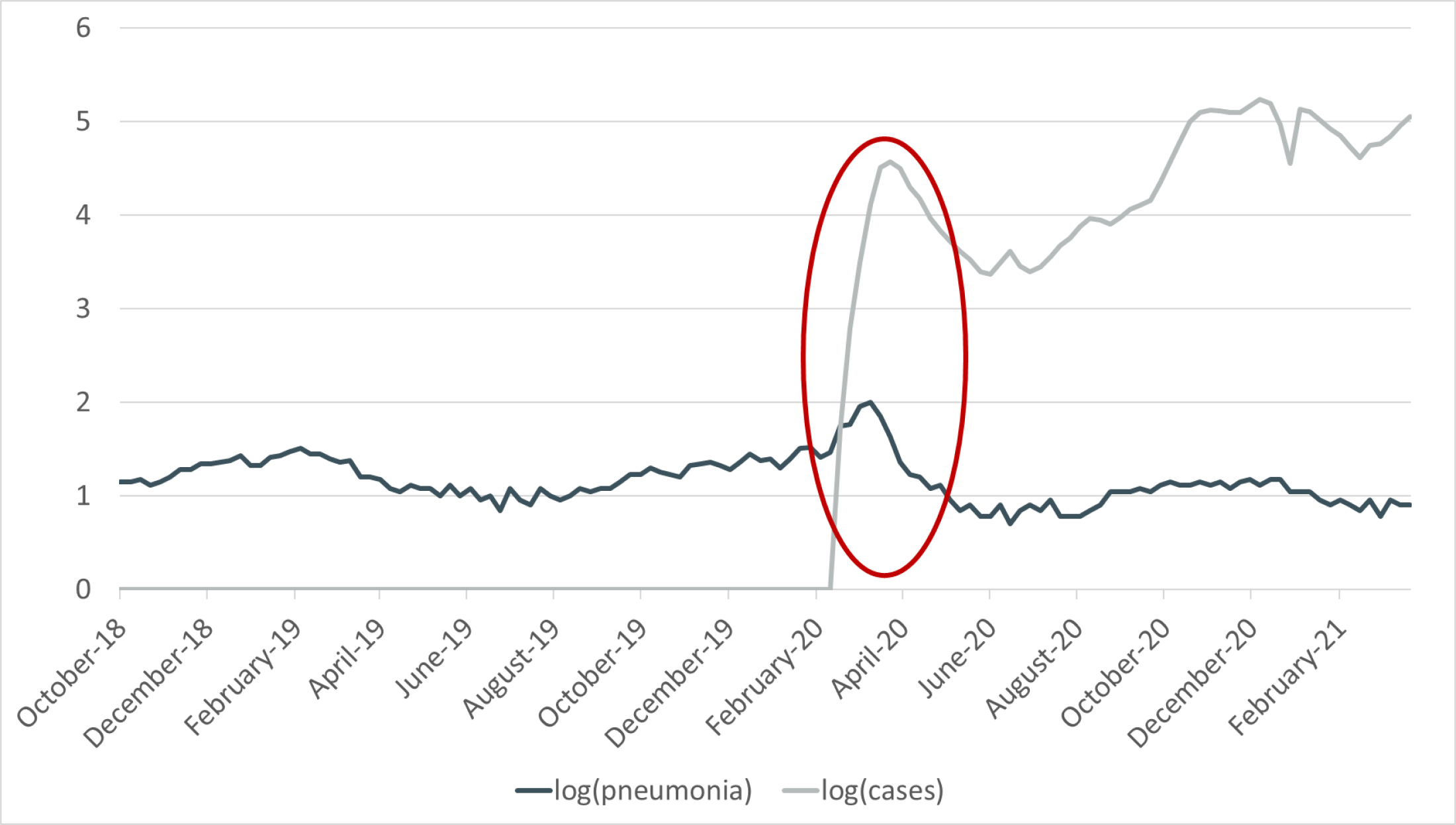

Supplement: S1 Fig — Note: Logarithm of search queries and cases used. (TIF) [file pone.0276485.s001.tif]

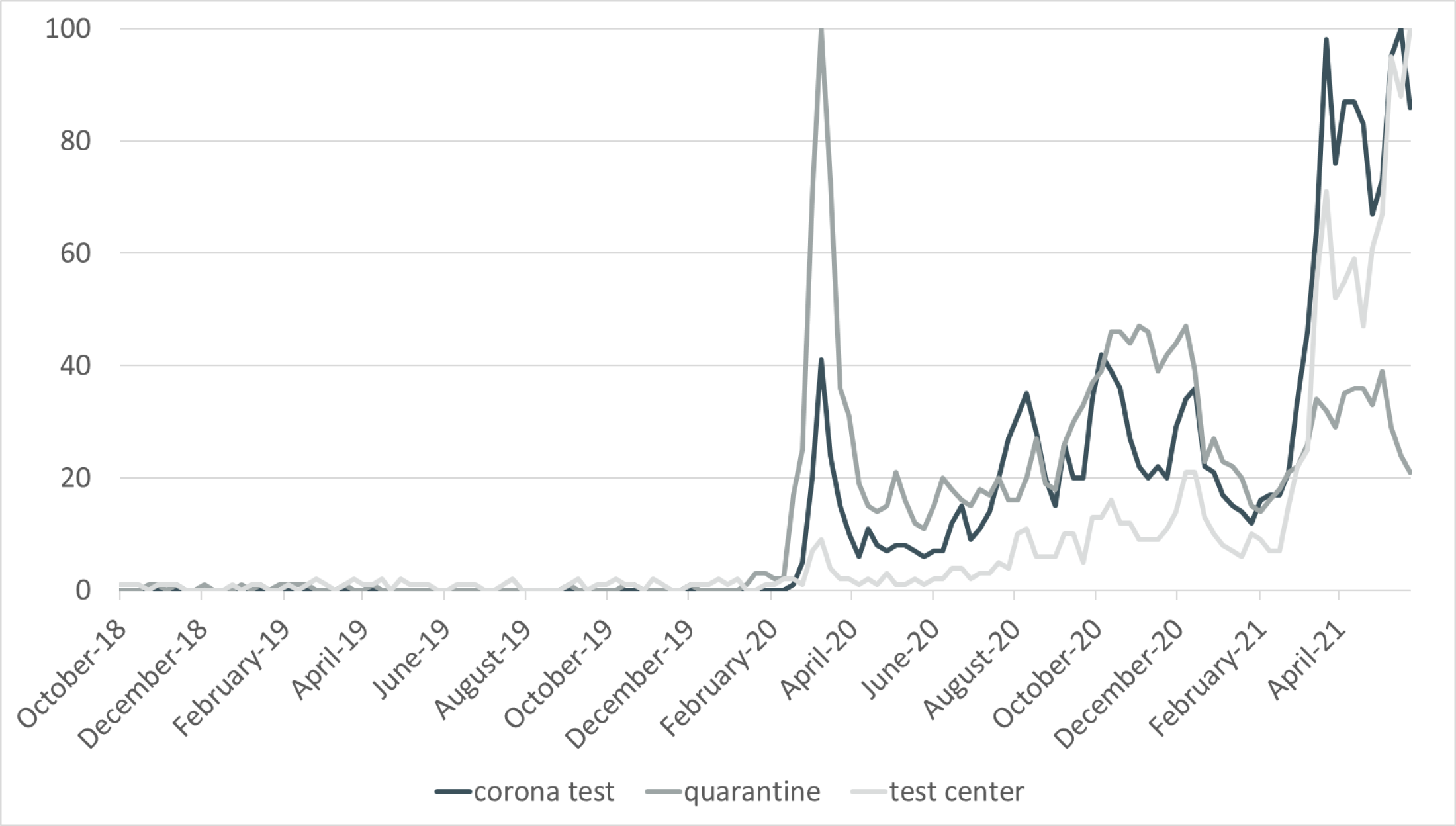

Supplement: S2 Fig — Note: The figures do not represent the ratio of search queries to one another, but the distribution of each search query separately over time. (TIF) [file pone.0276485.s002.tif]

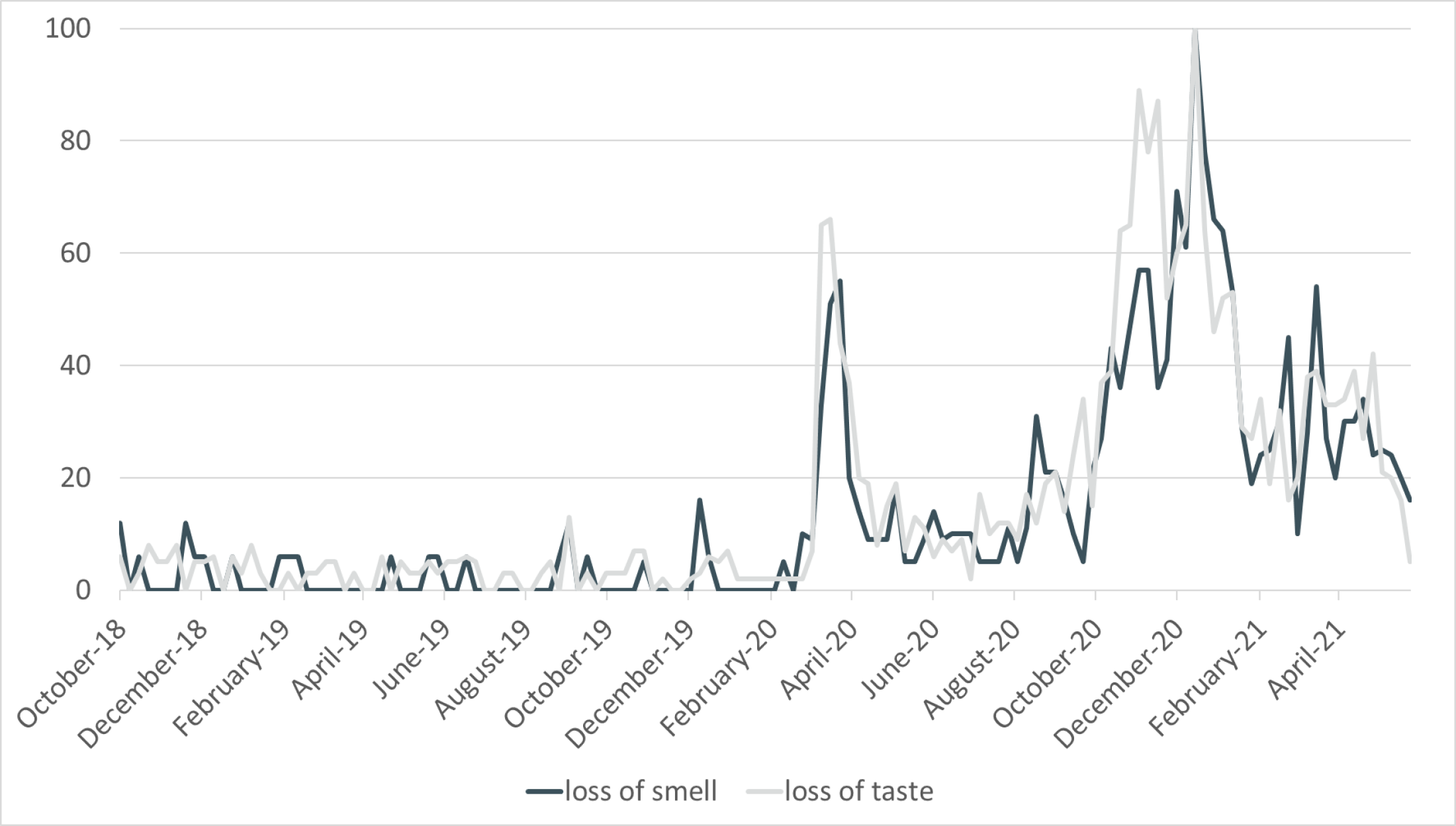

Supplement: S3 Fig — Note: The figures do not represent the ratio of search queries to one another, but the distribution of each search query separately over time. (TIF) [file pone.0276485.s003.tif]

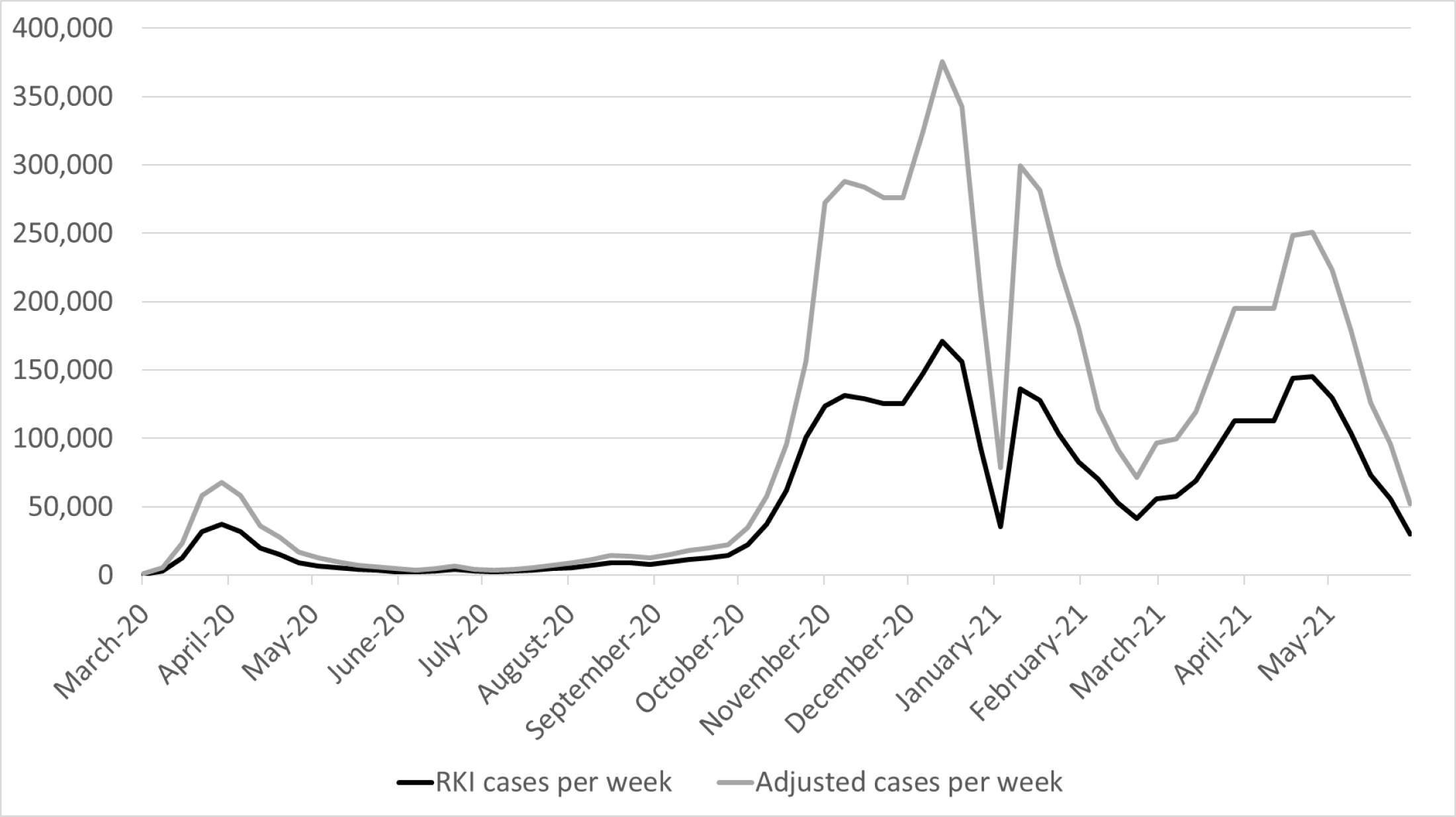

Supplement: S4 Fig — Note: Multiplier based on table, corrected for 20% asymptomatic cases. (TIF) [file pone.0276485.s004.tif]

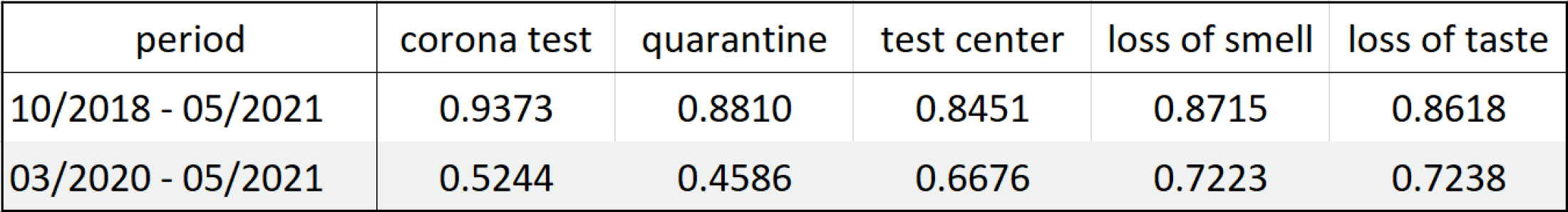

Supplement: S1 Table — Note: Table depicts the Spearman rank-order correlation coefficients. All values are significant at the 5% level. (TIF) [file pone.0276485.s005.tif]

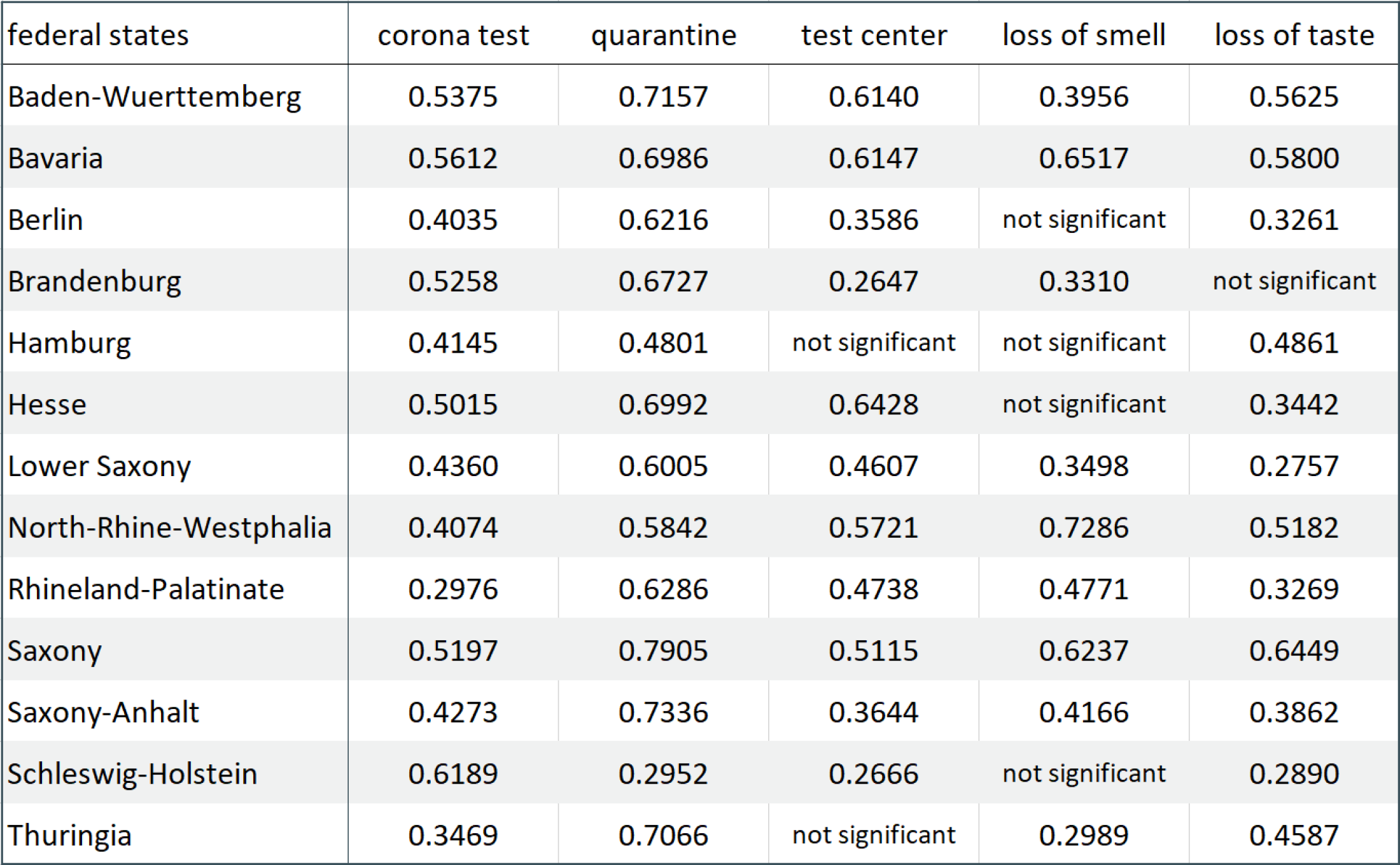

Supplement: S2 Table — Note: Table depicts the Spearman rank-order correlation coefficients. All values are significant at the 5% level. As case numbers per state are only available from May 10, 2020, we restrict our analysis to the period from that date until the end of May 2021. (TIF) [file pone.0276485.s006.tif]

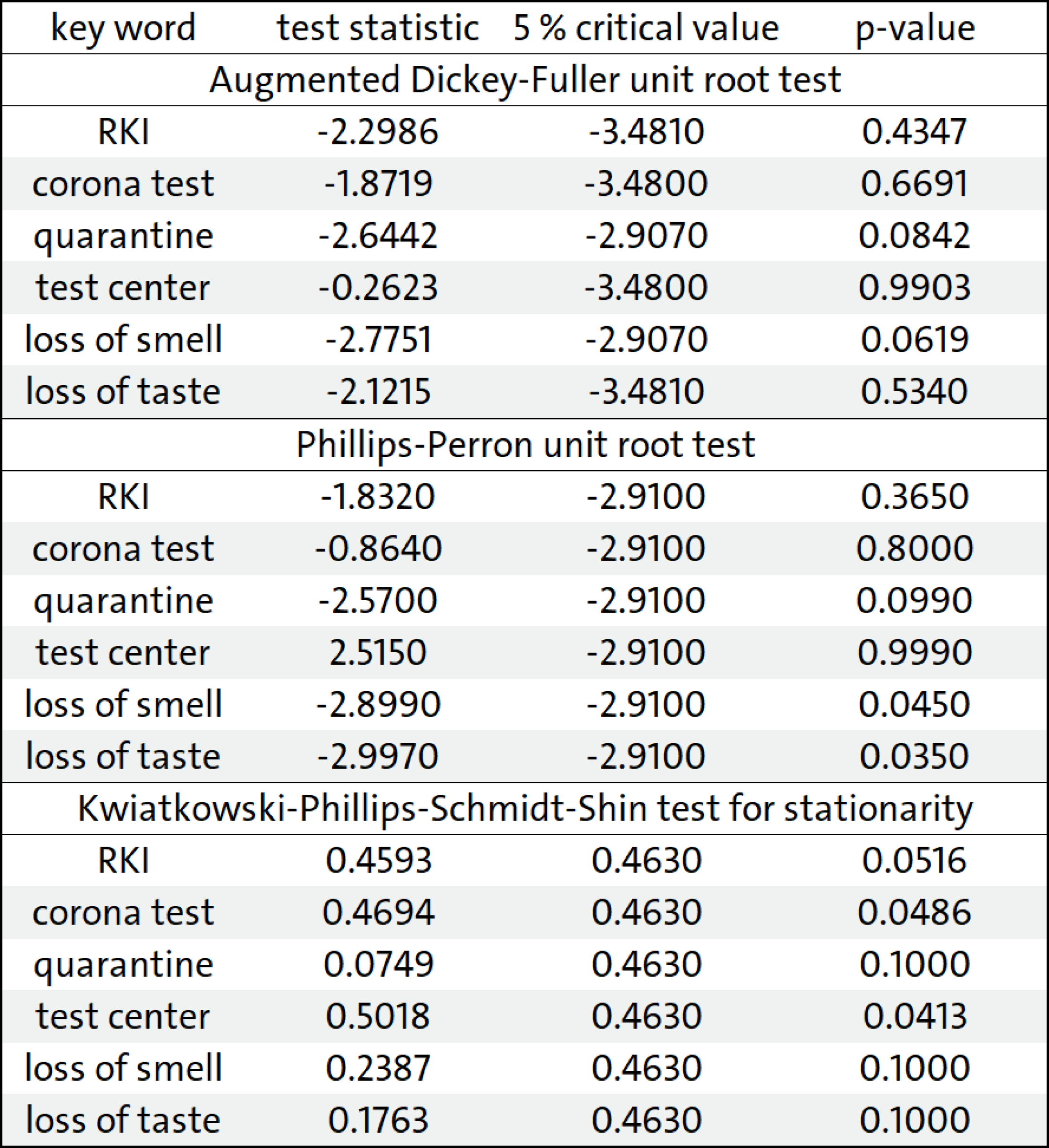

Supplement: S3 Table — Note: ADF: Augmented Dickey-Fuller, KPSS: Kwiatkowski-Phillips-Schmidt-Shin. Null hypothesis for ADF and Phillips-Perron: presence of a unit root (time series not stationary); for KPSS test it is stationarity. All tests are performed with the default of only adding a constant. (TIF) [file pone.0276485.s007.tif]

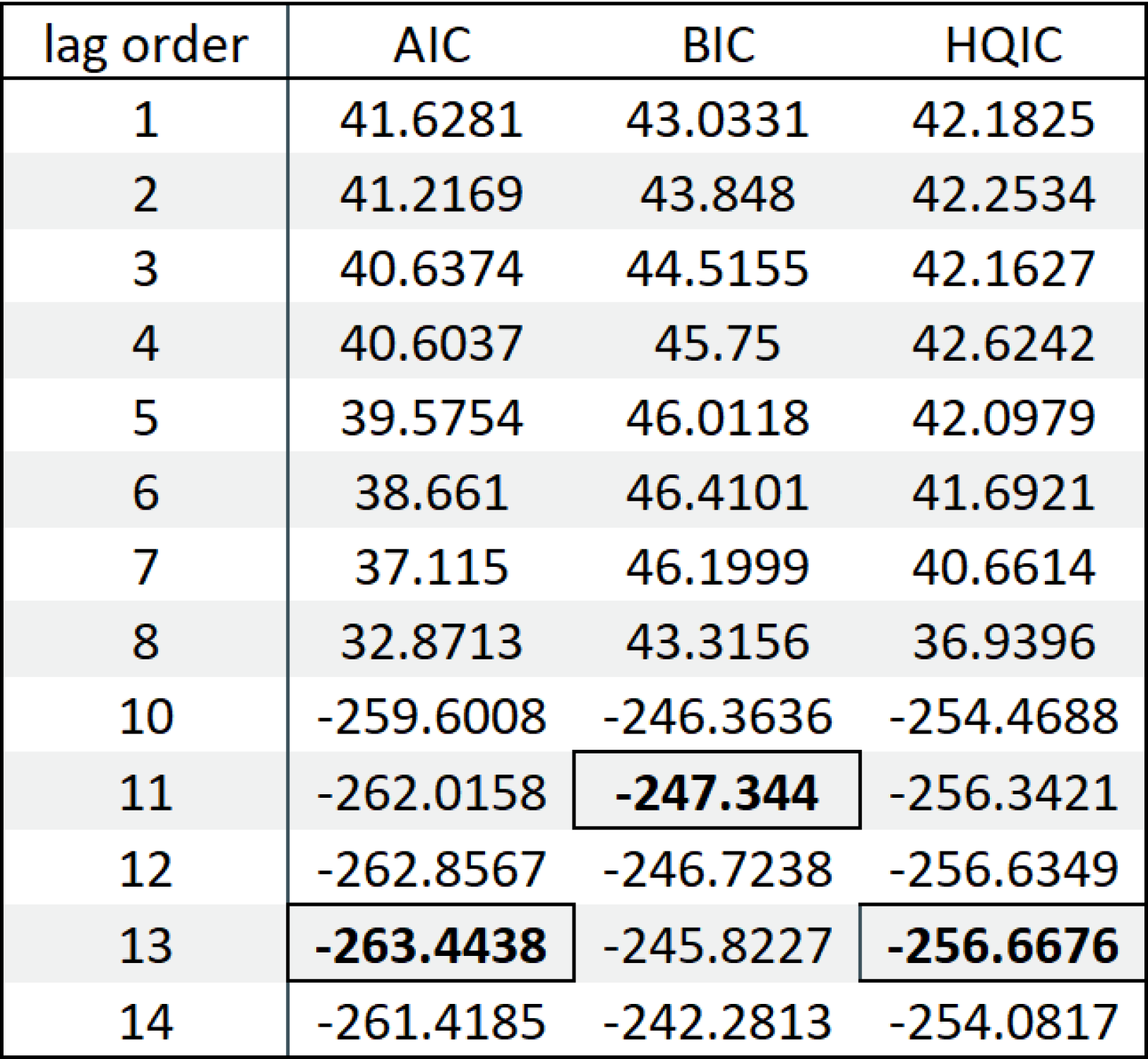

Supplement: S4 Table — Note: AIC: Akaike Information Criterion, BIC: Schwarz-Bayesian Information Criterion, HQIC: Hannan-Quin Information Criterion. (TIF) [file pone.0276485.s008.tif]

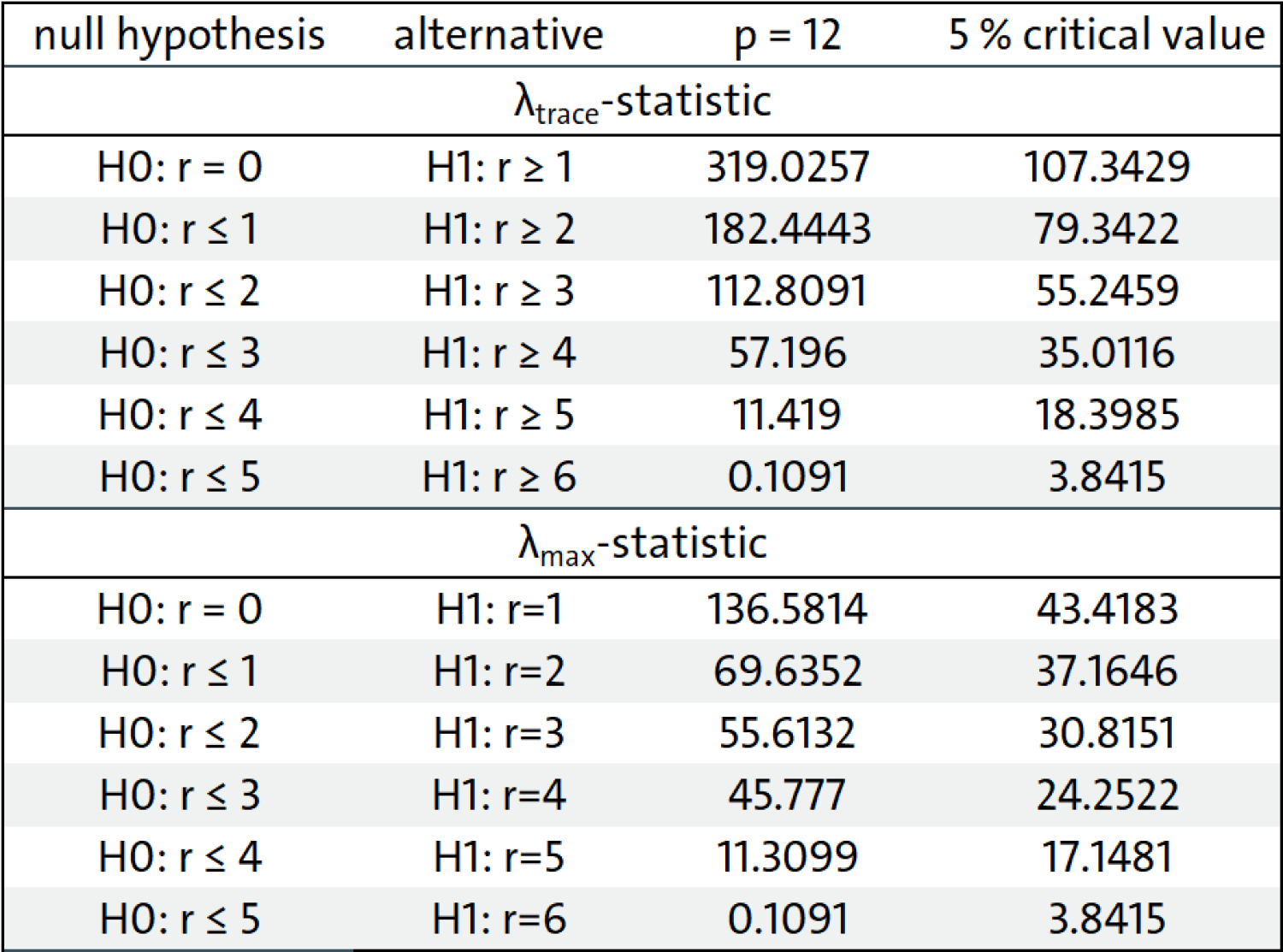

Supplement: S5 Table — Note: Table shows null hypothesis and alternative, the test statistics and 5% critical values for 12 lags for Johansen trace and maximum eigenvalue test for cointegration. (TIF) [file pone.0276485.s009.tif]

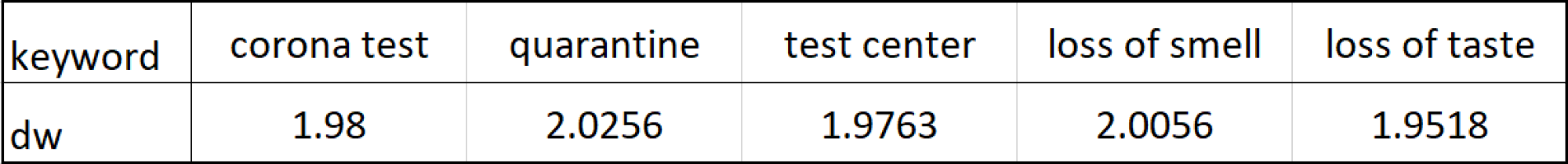

Supplement: S6 Table — Note: Table depicts Durbin Watson test statistics for p = 14 lags. (TIF) [file pone.0276485.s010.tif]
